# Subgroups of lifestyle patterns among hypertension patients: a latent-class analysis

**DOI:** 10.1186/s12874-018-0607-6

**Published:** 2018-11-12

**Authors:** Jalileh Ghanbari, Asghar Mohammadpoorasl, Leila Jahangiry, Mahdieh Abbasalizad Farhangi, Jamileh Amirzadeh, Koen Ponnet

**Affiliations:** 1 Health Education and Health Promotion Department, School of Public Health, Tabriz University of Medical Sciences, Tabriz, Iran; 2 Epidemiology and Biostatistics Department, School of Public Health, Tabriz University of Medical Sciences, Tabriz, Iran; 3 Tabriz Health Services Management Research Center, Tabriz University of Medical Sciences, Tabriz, Iran; 4 Drug Applied Research Center, Nutrition Research Center, Department of Community Nutrition, Tabriz University of Medical Sciences, Tabriz, Iran; 5 Department of Public Health, Health and Paramedical Faculty, Urmia University of Medical Sciences, Nazlou Campus, Urmia, Iran; 6 Department of Communication Sciences, Faculty of Political and Social Sciences, imec-mict-Ghent University, Ghent, Belgium

**Keywords:** Latent class analysis, Lifestyle pattern, Hypertension, Ageing

## Abstract

**Background:**

Hypertension remains one of the most important preventable risk factors for diseases and death. Identifying clustered patterns of modifiable lifestyle risk factors for hypertension and demographics factors related to these clustered patterns allows for targeting health prevention interventions. Therefore, this study aims to identify latent classes of hypertensive patients’ lifestyle risk factors based on the clustering of four modifiable lifestyle risk factors: eating, physical activity patterns, smoking habits, and blood pressure control.

**Methods:**

A total of 750 patients (*M*_age_ = 65.38 years, *SD*_age_ = 9.2 years) with diagnosed hypertension in urban and rural primary health care centers in Takab (Iran) were recruited randomly from August 2016 to February 2017. Latent class analysis was performed by using proc. LCA in SAS 9.2.

**Results:**

Three classes of lifestyle patterns were identified. About 14.4% of hypertensive patients were categorized in a low-risk class (I), 54.6% in an intermediate-risk class (II), and 31% in a high-risk class (III) of lifestyle. A one-year increase in age significantly increases the risk of membership in classes II and III. Similarly, being widowed or divorced increases the risk of membership in classes II and III. Also, having a higher education level decreases the risk of membership in classes II and III.

**Conclusions:**

This study contributes to the literature on lifestyle behaviors among older adults and provides evidence that there are considerable differences in lifestyle behaviors between subgroups of older adult patients. The three profiles of hypertensive patients’ conditions suggest that because behaviors often occur simultaneously within an individual level, a latent-class approach helps cluster co-occurrence risk behaviors and focuses on interventions targeted to several healthy behaviors among high-risk patients.

## Background

Hypertension remains one of the most important preventable risk factors for diseases and death. The prevalence of hypertension is increasing with age, and by the age of 60–69 years, more than half of a population has high blood pressure [1, 2]. The World Health Organization estimates that about one billion people suffer from hypertension worldwide and accounts for approximately 7.5 million deaths per year [3]. It is estimated that by 2025, 60% of adults will suffer from hypertension [4]. This represents a continuing trend in which the prevalence and absolute number of hypertensive patients has increased at a steep rate [3, 5]. Evidence indicates that hypertensive patients are at an increased risk of several chronic diseases, such as coronary artery diseases, stroke, and cardiovascular mortality [6], as well as some cancers [7]. Increased cardiovascular mortality in developing countries is largely due to modifiable risk factors, such as salt intake, smoking, physical inactivity, obesity, unhealthy eating habits, and in some cases, non-modifiable risk factors, such as age, gender, and heredity [8].

A systematic review of studies on hypertension among Iranian adults revealed that the prevalence of hypertension is 60% [9]. Recent evidence of the Framingham Heart Study indicates that systolic blood pressure is the most prevalent type of hypertension in patients aged 50 or above, so that by increasing the age, SBP increases by 10% per decade [10]. The pattern of diastolic blood pressure, however, differs with ageing, i.e. it increases until people are in the fifth decade, and slowly declines when people are over 60 years old [11].

Effective lifestyle modification programs and affordable strategies to control high blood pressure would benefit the at-risk populations [12]. The main causes of mortality in adults, especially in older adults, are that 53% of deaths are linked to unhealthy lifestyles and behaviors [13], like physical inactivity, tobacco smoking, poor diet, and excessive alcohol consumption. Most often, these unhealthy behaviors occur simultaneously or cluster within individuals [14, 15]. To date, the majority of studies focus only on changing a single unhealthy behavior. However, interventions that target these unhealthy behaviors simultaneously are necessary [16]. Traditional cluster and factor analyses are designed for continuous variables, but a latent-class analysis (LCA) is suitable for discrete and dichotomous variables. Hence, some studies report that an LCA seems to be logical and is a more informative approach for describing health behaviors [17, 18].

An LCA is a commonly used empirical approach to identifying subpopulations by shared item response patterns. It is also a commonly used data reduction tool for analyzing multivariate categorical data [18]. A finite number of exclusive classes of individuals categorized as latent variables is assumed to compose this population [19]. An LCA can be used to identify lifestyle indicators as being healthy or less healthy and can help inform preventive health efforts to modify disease outcomes. Only a few studies have applied an LCA to help investigate lifestyle risk factor clustering [17, 19, 20]. A recent study used an LCA approach to predict healthy lifestyle patterns among retirement-aged older adults [17]. The authors identified two classes of lifestyle patterns: healthy (53% men and 72% women) and less healthy lifestyles. They found there are meaningful gender differences in lifestyle behaviors among older adults.

Identifying the clustered patterns of modifiable lifestyle risk factors for hypertension and demographics factors related to clusters of unhealthy lifestyle factors allows targeting health prevention interventions. It is important, particularly among older hypertensive patients with respect to their individual barriers, to manage high blood pressure. Examining lifestyle risk factor clustering through an LCA for hypertensive patients may be relevant for guiding health prevention programs toward patients at a community level.

Therefore, the current study’s aim is to identify latent classes of hypertensive patients’ lifestyle risk factors based on the clustering of four modifiable lifestyle risk factors: eating patterns, physical activity patterns, smoking habits, and blood pressure control. We hypothesized that distinctive classes of major lifestyle patterns will be identified and that socio-demographic covariates, such as gender, would significantly predict the identified classes. Identifying the classes may indicate and inform who may be at risk of unhealthy lifestyle patterns.

## Methods

### Design and participants

This cross-sectional study was conducted in Takab, a county with an estimated population of 81,000 people and that is located in the West Azerbaijan province of Iran. A total of 750 patients with diagnosed hypertension in Takab’s urban and rural primary health care centers were randomly recruited from August 2016 to February 2017. The inclusion criteria were 1) having diagnosed hypertension in the previous 6 months, 2) being aged 50 years and older, 3) living in Takab, and 4) having household health records in health care centers. Exclusion criteria were 1) having diabetes, 2) having cognitive disorders, and 3) having no interest in participating in the study.

Hypertensive patients aged 50 years and older from Takab’s urban and rural regions were invited to participate in the study. During an Iranian diseases prevention program, all women and men were screened in Iranian Women Health Services (SABA; Persian name of program) and Iranian Men Health services (SAMA, Persian name of program) respectively. As such, all patients’ health records were available in the health care centers. Patients with diagnosed hypertension were randomly selected and contacted by telephone. During the phone interview, eligible interested patients were asked to refer to health care centers for clinical assessment by trained health care researchers. In total, 750 patients completed the clinical assessment from the 900 invited patients (a response rate of 83.3%).

### Latent-class indicators

In this analysis, the underlying latent variable is healthy lifestyle patterns. Four indicators covering dietary patterns, physical activity, tobacco use, and high blood pressure control were selected. The indicators were chosen to ensure conditional independence of variables within each latent class [21]. Binary indicators (healthy versus unhealthy behavior) were created based on existing recommendations, as described below. The four binary indicators resulted in 24 possible response patterns.

### Dietary patterns

Dietary intake was measured using a 147-item food frequency questionnaire (FFQ) [22] originally developed for the Tehran Lipid and Glucose Study (TLGS, Research Institute for Endocrine Sciences in Iran). The FFQ is a Willett-format questionnaire that has been adapted to the Iranian context and that includes questions about average consumption and frequency of 147 food items during the past year.

The Dietary Approaches to Stop Hypertension (DASH) adherence score was calculated based on dietary information from the FFQ questionnaire. This was a composite score, composing of subscores from seven food and nutrient components (grains and legumes, fruits and vegetables, nuts and seeds, dairy, meat, fats, and sodium). A score of 0 to 1 was given to each component based on intake compared to the intake recommendation, yielding a maximum total score of seven. We considered having six or more DASH diet elements indicated a healthy DASH diet, having 4–5 DASH diet elements was an intermediate DASH diet, and having? 3 DASH diet element was a poor DASH diet [23].

### Physical activity

Walking, sitting, and engaging in moderate- and vigorous-intensity physical activities during the 7 days prior to the interview were measured using the International Physical Activity Questionnaire (IPAQ-short form), a well-validated questionnaire in Iran [24]. The total physical activity score was calculated by summation of the duration (in minutes) and frequency (days) of walking and moderate-intensity and vigorous-intensity activities. In the present analyses, we used the metabolic equivalent (MET) (kcal/kg-hour) to measure an activity’s intensity and energy expenditure in kilocalories. The metabolic equivalent score of an activity done by a participant was calculated as the product of the activity’s MET and duration. A participant’s total metabolic equivalent score (METS) was calculated by summing the products across all activities. Total physical activity (PA) was classified based on METS into two subgroups: METS < 600 and METS? 600 [25].

### Smoking

One question about smoking was included in the sociodemographic characteristics’ section, and a binary variable for smoking was created (never smoked versus current and former smokers).

### Hypertension control

According to the European Society of Hypertension and the European Society of Cardiology, a target of < 140/90 mmHg for hypertensive patients is recommended [16].

### Anthropometric measurements

Blood pressure was measured with a mercury sphygmomanometer twice in the same arm after the individual seated at rest for 10–15 min [1]. The individuals’ weights—while the individuals were dressed in light clothing and without shoes—were measured using a calibrated scale (Seca, Hamburg, Germany model 8,811,021,658) to the nearest 0.1 kg. Height was measured without shoes using a stadiometer (Seca, Hamburg, Germany) to the nearest 0.1 cm [26]. The body mass index (BMI) was calculated by dividing one’s individual’s weight in kilograms by the square of one’s height in meters [27]. Waist circumference was measured to the nearest 0.1 cm with a measuring tape spanning the midpoint between the last rib and the iliac crest, with the subjects standing and breathing normally. Hip circumference was measured at the maximum level over light clothing using a non-stretchable tape measure, without any pressure against the body’s surface. Measurements were recorded to the nearest 0.1 cm [28].

### Statistical analyses

An LCA is a latent categorical variable model, and it classifies homogeneous individuals. An LCA model was performed using 4 indicator variables: physical activity)2 response categories), dietary patterns (3 response categories), hypertension control (2 response categories), and smoking status (2 response categories) [29].

The descriptive continuous variables were analyzed using a one-way analysis-of-variances, and categorical variables were analyzed using a Chi-square test. The LCA analyses were conducted by using a proc. LCA in SAS 9.2 software. The LCA outcomes include the number of latent classes, the probability of each indicator in each class, and the classification of individuals based on their most likely latent-class membership. Due to various iterations for the number of the latent variable’s identified classes, and by comparing the observed response patterns’ frequencies with the expected response patterns, the LCA determines the best model and calculates a statistic similar to χ^2^, called G2. Based on the G2 statistic, Akaike information criterion (AIC) and Bayesian information criterion (BIC) can be calculated for model selection. For all information criteria, a smaller value represents a more optimal balance of model fit and parsimony; thus, a model with the minimum AIC or BIC might be selected [30–32]. For performing an LCA, four dichotomous observable variables (i.e., indicators) were used for assessing lifestyle behaviors as latent variables. After finalizing the model, an individual’s age, marital status, and educational level were examined as covariates.

## Results

The participants’ descriptive characteristics are presented in Table 1. The mean age of the hypertensive patients was 65.3 years (*SD* = 9.2). Women were significantly younger than men (at 63.3 years [*SD* = 9.2] and 68 years [*SD* = 9.7], respectively). The majority of participants were female (56.1%), illiterate (73.1%), and married (77.2%). Women were significantly less educated than men. The mean BMI was 26.8 kg/m^2^ (*SD* = 9.2). Women had a significantly greater BMI than men (27.5 kg/m^2^ and 25.8 kg/m ^2^, respectively).

**Table 1:** Descriptive characteristics of patients with hypertension

	Men (*n* = 329) N (%)/mean [SD]	Women (*n* = 421) N (%)/mean [SD]	Total (*n* = 750) N (%)/mean [SD]	*p*-value
Age in years	68 [9.7]	63.3 [9.2]	65.38 [9.2]	<.0001
Education				<.0001
Illiterate	195 (35.6%)	353 (64.4%)	548 (73%)	
Elementary	122 (37.08%)	65 (15.43%)	187 (24.9%)	
High school and university	7 (2.12%)	2 (0.47%)	9 (1.2%)	
Marital status				.597
Married	257 (44.4%)	322 (75.05%)	579 (77.2%)	
Single	72 (21.88%)	99 (23.51%)	171(22.8%)	
Body mass index	25.8 [3.8]	27.5 [4.95]	26.8 [4.6]	<.0001
Systolic BP	137.3 [15.2]	137.8 [17.6]	137.6 [16.6]	.680
Diastolic BP	85.2 [9.8]	85.8 [11.3]	85.5 [10.6]	.438
WC	94.2 [10.95]	95.2 [13.7]	94.8 [12.6]	.285

*BP* Blood pressure *WC* Waist circumference

According to the task force for the arterial hypertension management of the European Society of Hypertension [15], about 43% of the participants had controlled hypertension. Based on the physical activity guideline, only 36.3% of the patients reached *PA*? 600 MET minutes/week; however, men had significantly higher PA levels than women. Also, only 8% of patients had the optimal adherence to DASH diet intake (having? 6 DASH elements).

### Latent-class findings

The model-fit indices are shown in Table 2. Based on the four dichotomous indicators, there were 24 possible response patterns. We attempted to fit the LCA models with classes ranging from 1 to 5. For each LCA model, the G2, AIC, and BIC were computed (Table 3). According to the model’s indices and the model’s results, we concluded that a three latent-class model was appropriate for the patients. The results of the three LCA model showed that differences between the expected and observed response pattern frequency were not statistically significant (*G2* = 4.89, *df* = 6, *p* = .557).

**Table 2:** Comparison of LCA models with different latent classes based on model selection statistics

No. LCA	No. parameters estimated	Loglikelihood	G^2^	df	AIC	BIC	*P*-value
1	5	− 1709.6	22.65	18	32.65	55.75	.232
2	11	− 1783.4	8.20	12	30.20	81.02	.769
3	17	− 1781.7	4.89	6	38.89	117.43	.557
4	23	− 1780.2	1.88	0	47.88	154.14	–

*Df* degrees of freedom, *AIC* Akaike information criterion, *BIC* Bayesian information criterion

**Table 3:** Prevalence of each lifestyle behavior indicators used in the latent class analysis among patient with hypertension

Items	Men (*n* = 329) *n* yes (%)	Women (*n* = 421) *n* yes (%)	Total (*n* = 750) *n* yes (%)	*p*-value
Smoking	108 (32.8%)	22 (5.22%)	130 (17.3%)	< .0001
Uncontrolled hypertension	241 (73.3%)	186 (44.2%)	427 (56.9%)	.846
PA < 600 MET-minutes/week	191 (58.1%)	287 (68.2%)	478 (63.7%)	.004
Dash based diet				.209
Having ≤3 dash elements (I)	80 (24.3%)	118 (28.02%)	198 (26.4%)	
Having 4–5 dash elements (II)	226 (68.7%)	264 (62.7%)	490 (65.3%)	
Having ≥6 dash elements (III)	23 (7%)	39 (9.3%)	62 (8.3%)	

*PA* Physical activity, *MET* metabolic equivalent score

Latent class membership and response probabilities for each indicator and covariates are summarized in Table 4. The probability of membership in each latent class is shown in the first row of Table 4. About 14.4% of hypertensive patients were categorized in the low-risk class (I), 54.6% in the intermediate-risk class (II), and 31.0% in the high-risk class (III) lifestyles. These probabilities form the basis for interpreting and labeling the latent classes.

**Table 4:** The three latent classes model of lifestyle behaviors and its covariates among patients with hypertension

	Low risk lifestyle - Class I	Intermediate risk lifestyle – Class II	High risk lifestyle – Class III
Latent class prevalence	0.144	0.546	0.310
Items			
Smoking			
No	1.000	0.781	0.827
Yes	0.000	0.219	0.173
Hypertension un-controlled			
No	1.000	0.347	0.313
Yes	0.000	0.653	0.687
Physical inactivity			
No	0.408	0.367	0.334
Yes	0.592	0.633	0.666
Nutrition status			
Having ≥6 dash elements (III)	0.046	0.000	0.245
Having 4–5 dash elements (II)	0.735	1.000	0.005
Having ≤3 dash elements (I)	0.219	0.000	0.750
Covariates (odds ratio)		OR	OR
Age (*p* 0.033)	Ref	1.12	1.12
Marital status (single) (*p* .089)	Ref	7.23	5.18
Education (*p* 0.009)	Ref	7.14	0.77

The conditional probabilities of a “Yes” response to each risk behavior for lifestyle are listed in Table 4. The probability of a “No” response can be calculated by subtracting the item response probabilities from 1. Larger conditional probabilities are in bold to highlight the overall pattern. Latent class III was characterized by a high probability of responding “Yes” to nearly all of the risk-taking behaviors. Individuals in this latent class were likely to report that they had engaged in the study’s listed risk-taking behaviors, including uncontrolled blood pressure (BP), inactivity, and adherence to? 3 DASH diet elements. In contrast, those in latent class I were likely to report not having smoked, maintaining a controlled BP, being inactive, and using more than six DASH diet elements.

The odds ratio indices of membership in each class—compared to the first class—and associated with the independent variables are also listed in Table 4. Membership in classes II and III increases with a one-year increase in age. Membership in classes II and III are also increased by being widowed or divorced. Membership in classes II and III are, however, decreased with a higher education.

## Discussion

This study provides new evidence about the lifestyle patterns of patients with hypertension and identified subgroups of older adults with respect to their patterns of eating, smoking, maintaining physical activity, and ensuring hypertension control. The findings suggest a divergence in risk behaviors within hypertensive patients.

Four primary results are evident from this research. First, we identified three distinct profiles of hypertensive patients’ lifestyle conditions, including a low-risk lifestyle (14.4%), an intermediate-risk lifestyle (54.6%), and a high-risk lifestyle (31%). Second, class III was distinguished as high-risk class for lifestyle of hypertensive patients. Overall, hypertensive patients in class I are people with controlled BP (100%) and who do not smoke cigarettes (100%), who are not inactive (41%), and who use 4–5 DASH diet elements (73%). Third, hypertensive patients in class II are people with un-controlled BP (65%) and who smoke cigarettes (22%), are inactive (63%), and use 4–5 DASH diet elements (100%). Fourth, hypertensive patients in class III are people with un-controlled BP (68.7%) and who smoke cigarettes (17%), are inactive (66%), and use fewer than three DASH diet elements (75%).

The three profiles of hypertensive patients’ conditions for lifestyle and behavioral risk factors suggest the need to treat common patterns of patients with hypertension simultaneously, thereby improving efficiency and cost-effective interventions. In two classes, most of the patients tended to have un-controlled hypertension and did not adhere to their medicines. Targeting high BP delivery services and more frequent and opportunistic BP testing are needed by the health care workforce, including primary care physicians, pharmacists, nurses, and other allied health professionals, as well as in primary health care settings [33]. Also, our results suggest a recommendation that regular screening and surveillance programs should be tailored and developed for older adult hypertensive patients.

It seems that for the control of hypertension among older adult patients, the fragmentation of health care for patients according to their lifestyle classifications appears to be an important contributor. To bring hypertension under control, simultaneous efforts focused on educating patients and providers about hypertension [34], developing efficacious primary and secondary prevention, and implementing lifestyle change interventions in people at elevated risk can help reduce a large proportion of mortalities related to hypertension [35].

According to our results, a clear distinction between groups was evident, particularly with respect to dietary patterns, physical activity, and hypertension control. Class I was distinguished from class II and class III as being the low-risk lifestyle with optimal BP control. Respondents who belong to class I tend to control their BP by medications and optimum diets and avoid smoking. Therefore, the probability of reaching recommended BP control was low in both classes II and III. The dietary indicators showed similar probabilities in classes I and II for having 4–5 DASH diet elements. Overall, regardless of latent-class analysis, the probability of reaching the recommended DASH diet intake was low across the whole sample. Södergren et al. [17] identified two classes of lifestyle when they used latent-class lifestyle modeling among older adults. They concluded that fruit intake was a good indicator distinguishing the “healthier” class, whereas consumption of vegetables and fast food (by women) could not clearly distinguish older adults in the other two classes. This is consistent with our data, showing an overall low consumption of vegetables and fruits compared with the recommended level among older adults. Similar results were obtained by Sodergren et al. [36]. DASH diet is the most effective diet for hypertension control and emphasizes fruits, vegetables, whole grains, low-fat dairy products, and products reduced in fat and cholesterol, with a low intake of red meat [37, 38].

Though less than one-fifth of the patients belonged to class I, they had relatively more probability of engaging in healthy behaviors related to hypertension control. For example, they have kept their blood pressure under control, adhered more to a DASH-style diet, and did not smoke. However, the vast majority of the participants had a lower probability of achieving the recommended level of physical activity. In particular, all of the groups shared similarly high probabilities of inadequate physical activity. Physical activity is commonly recommended as an important lifestyle modification that may aid in hypertension prevention [39, 40]. Studies have shown that physical activity is a main predictor of progression from prehypertension to hypertension [41]. Whelton et al. found that exercise produces substantial improvements in systolic and diastolic blood pressure among older adults [42]. They also suggested that body composition improvements from physical activity are associated with BP reductions and improve cardiovascular health in older adults. A recent meta-analysis of 27 randomized trials documented a 4-mmHg reduction in systolic BP among older and middle-aged adults from an aerobic exercise intervention. Indeed, our findings are consistent with the physical activity guidelines that exercise of 30 min or more per day might reduce or control hypertension [36, 42].

It seems that clustering and identifying co-occurrence risk behaviors of an at-risk population may help prevent and lead to co-change, which is one of the most effective approaches in preventing high-risk behaviors [21].

It can be expected that older adult patients encounter multiple challenges and barriers to performing exercise and physical activity. In a study by Moschny et al., poor health emerged as the most important barrier to sufficient physical activity, and low perceived physical abilities were strongly associated with lower physical activity among older adults [43].

The majority of the participants in our study were illiterate, and a higher proportion of respondents did not adhere to the pattern of healthy lifestyles and physical activities. The results suggest that having a higher education level decreases the risk of membership in classes II and III. Consistent with our results, Baker et al. found that higher education was related to belonging to the healthiest class (2011) [44]. It can be concluded that lower education is associated with an unhealthy lifestyle pattern. Also, a 1 year increase in age increases the risk of membership from latent class I to class II and class III. Similarly, being married decreases the risk of membership in classes II and III seven times compared to divorced or widowed patients. Studies about healthy behavior by marital status has consistently identified that single individuals generally report poor health and have a higher mortality risk than their married counterparts [45].

To our knowledge, no research has investigated lifestyle pattern among hypertensive patients. However, one limitation of our study is that smoking as a criterion for clustering might not be a good indicator for predicting behavioral patterns in patients. Also, alcohol consuming among Iranian population is not possible to measure because people will not reply in a reliable and trustworthy way. Furthermore, it might be interesting for future studies to include other factors related to hypertension, like alcohol use.

## Conclusions

Because behaviors often occur simultaneously within an individual, a latent-class approach might help cluster co-occurrence risk behaviors and focus on interventions targeting the several healthy behaviors among high-risk patients. The LCA assists investigators in identifying simple, latent-class patterns within complicated observed categorical indicators. Our study considers the subgrouping of an older adult patient sample into three classes. Results show a considerable percentage of patients in particular have uncontrolled hypertension. In addition, we found that marital status is more likely to be a preventive factor for unhealthy behavior. These analyses provide important insights into how we might target lifestyle-promoting strategies among older adult hypertensive patients. However, more research is needed to understand the modifiable determinants of these behavioral patterns so proper services can perform.

## Abbreviations

BMI: Body mass index
DASH: Dietary approaches to stop hypertension
FFQ: Food frequency questionnaire
IPAQ: International physical activity questionnaire
LCA: Latent class analysis
MET: Metabolic equivalent
PA: Physical activity
